# Metabolomics analysis reveals the metabolite profiles of *Rheum tanguticum* grown under different altitudinal gradients

**DOI:** 10.1186/s12870-024-04933-9

**Published:** 2024-03-28

**Authors:** Lingling Wang, Shuo Zhao, Jianan Li, Guoying Zhou

**Affiliations:** 1 Key Laboratory of Tibetan Medicine Research, Northwest Institute of Plateau Biology, Chinese Academy of Sciences, Xining, 810008 China; 2 Resource Institute for Chinese and Ethnic Materia Medica, Guizhou University of Traditional Chinese Medicine, Guiyang, 550025 China; 3 University of Chinese Academy of Sciences, Beijing, 100049 China

**Keywords:** Altitudinal gradient, Environmental influence, Mechanisms, Metabolomics, *Rheum Tanguticum*

## Abstract

**Background:**

Plant growth and quality are often affected by environmental factors, including geographical location, climate, and soil. In this study, we describe the effect of altitudinal differences on the growth and active ingredients in *Rheum tanguticum* Maxim. ex Balf. (*R. tanguticum)*, a traditional Chinese medicinal herb known for its laxative properties.

**Results:**

The results showed that plants grown at lower altitudes had better growth performances than those in higher altitude areas. The yield varied by 2.45–23.68 times with altitude, reaching a maximum of 102.01 t/ha. In addition, total anthraquinone and total sennoside contents decreased with increasing altitude, whereas total tannins increased with increasing altitude. The total anthraquinone content of the indicator compound reached 5.15% at five experimental sites, which exceeded the Chinese Pharmacopoeia standard by 70.87%. The content of the other two categories of active ingredients reached a maximum value of 0.94% (total sennosides) and 2.65% (total tannins). Redundancy analysis revealed that annual rainfall, annual average temperature, annual sunshine hours, and pH significantly affected growth and active ingredients. Moreover, key metabolites, such as flavonoids, amino acids and their derivatives, phenolic acids, lipids, and terpenes, were differentially expressed between samples from low- and high-altitude cultivation areas. These metabolites were enriched in the flavonoid and flavonol biosynthetic pathway and the monoterpene biosynthetic pathway.

**Conclusions:**

These results suggest that high anthraquinone content was observed in the lowest-latitude cultivation area due to low rainfall and alkaline soil pH. Key metabolites were significantly upregulated in high-latitude cultivation areas. These results provide a scientific basis for quality control and the systematic cultivation of *R. tanguticum*.

**Supplementary Information:**

The online version contains supplementary material available at 10.1186/s12870-024-04933-9.

## Introduction

Rhubarb is a perennial herb in the family Polygonaceae [1]. Its dried roots and rhizomes are used as medicine and have been shown to be effective in purging heat, dredging intestines, cooling blood, detoxifying, removing blood stasis and stimulating menstruation [2]. Rhubarb’s medicinal value has been documented in traditional Chinese medicine (TCM) classics, such as Treatise on Febrile Diseases and Miscellaneous Diseases and Four Medical Codes [3]. The results of modern pharmacological studies have revealed some of its other activities, including anti-inflammatory and antibacterial activity, as well as its ability to lower blood fat levels and protect the kidneys [4, 5]. Rhubarb has been widely used for thousands of years in China and many countries around the world and has been included in the national pharmacopoeias of China, the United States, Japan and other countries [6]. *Rheum tanguticum Maxim. ex Balf.* (*R. tanguticum*), one of the three “genuine rhubarb” species recorded in the Pharmacopoeia [7], is mainly distributed in Sichuan, Gansu, Qinghai and other places that is endemic to the Qinghai-Tibetan Plateau. The climate and geographical characteristics of the Qinghai-Tibet Plateau endow *R. tanguticum* with precious bioactive compounds. The distinctive pharmacological properties of *R. tanguticum* have made it widely popular in in the market. Due to the increasing market demand and the negative impact on natural populations, efforts have been made to cultivate *R. tanguticum* in the Qinghai-Tibet Plateau region. This become an important source of revenue for farmers,, especially for high-altitude areas where agricultural outputs were low and options for income generation were largely dependent on natural resources.

In contrast than wild products, the yields of cultivated plants are able to consistently meet market demands and protect wild *R. tanguticum* resources. However, the quality of cultivated products from different origins varies due to differences in geography, harvesting years, and processing methods [8]. Xiong et al. studied the metabolites of *R. tanguticum* in canopy-covered and open habitats and found that different habitats and ecotypes result in differences in targeted metabolites. *R. tanguticum* grown in understory environments exhibits better medicinal properties [9]. In another study on the composition of *R. tanguticum* from different sources, results indicated that *R. tanguticum* samples from Qinghai had higher levels of active ingredients than those from Gansu and Sichuan provinces [10]. It has been suggested that the anthraquinone content of two-year-old *R. tanguticum* reached the standard of the Chinese Pharmacopoeia. At three years old, the anthraquinone content was significantly higher than that of two-year-old *R. tanguticum* [11]. Therefore, effectively distinguishing *R. tanguticum* from different cultivation regions is imperative for accurate quality control of its products. Metabolomics can be used to effectively address this problem through comprehensive analysis of plant extracts and lead to the overall evaluation of the quality of traditional Chinese medicinal products [12]. Previous studies have used widely targeted metabolomics to decipher changes in nutrient composition in plants grown under different environments [13]. Li et al. [14] applied wide-target metabolomics to reveal changes and the dynamic accumulation of metabolites in walnut and hickory seed coats, while Lin et al. accurately identified differentially abundant metabolites in aconite cultivated in Anxian and Jiangyou by using metabolomics [15]. These differential compounds contribute to origin tracing and quality identification in aconite. With the widespread adoption of UPLC-Q-TOP/MS technology and its integration with multivariate statistical methods such as partial least squares discriminant analysis, metabolomics techniques can be better used to identify differential substances among samples. UPLC‒MS/MS is considered a feasible approach for the accurate quantification of the quality of *R. tanguticum* cultivated in different regions. *R. tanguticum* grows in areas at altitudes of 1800–4300 m, and the cultivation environment is closely related to its growth performance and quality [10]. The soil, temperature, moisture and light characteristics prevalent during the cultivation of medicinal materials leads to differences in growth forms and in the chemical composition of target plants. Liu et al. [16] found that high-altitude environments significantly increase the yield and quality of *Pseudostellaria heterophylla*. In another study, Nadeem et al. [17] found a negative correlation between plant morphological characteristics, including height, stem diameter and leaf area, and elevation in ​​*Podophyllum hexandrum Royle*. Worku et al. [18] analyzed the effect of altitude on the biochemical composition of *Coffea arabica* L. and found that caffeine and chlorogenic acid contents decreased with increasing altitude. Although there have been studies on cultivation techniques such as fertilization and density for *R. tanguticum*, as well as the application of metabolomics to explore differential compounds under different treatments, further research is needed to investigate the effect of altitude on the growth and pharmacological activity as well as metabolite profiles of *R. tanguticum*.

The aim of this study was to evaluate the effect of altitudinal gradients on growth indicators and the active ingredient content of *R. tanguticum*. We employed widely targeted metabolomics analysis to analyze differentially abundant metabolites and enriched pathways in *R. tanguticum* across different altitudes and then applied a redundancy analysis method to reveal potential relationships among growth characteristics, active ingredients, metabolites and environmental factors. Through evaluating the phenotype and components of *R. tanguticum*, we can uncover its growth pattern at altitude. So as to achieve the goal of improving the quality of medicinal materials.Taken together, the results of this study provide a theoretical basis for evaluating quality and cultivation site selection for *R. tanguticum.* This contributes to the protection of wild resources, maintains ecological balance, and provides sustainable economic benefits to farmers.

## Results

### Effect of altitudinal gradients on the growth characteristics of *R. tanguticum*

The interannual variability in yields across the same test sites is outlined in Table 1. In summary, the yield increased with increasing age, except in Datong (DT), with the lowest yield recorded in 2-year-old plants (17.76, 10.84, 7.05, 3.64 and 0.75 t/ha). The value reached a maximum in 4- and 5-year-old plants (102.01, 78.87, 64.86, 42.84 and 41.69 t/ha), albeit with no statistically significant differences. Under the same age, *R. tanguticum* yield decreased with increasing altitude. Among the 5 experimental sites, Guoluo (GL) had the highest altitude and the lowest yield between 2 and 5 years, while the opposite was true for Ledu (LD).

**Table 1:** Comparison of the yield (t/ha) of *R. tanguticum* at different ages at the same experimental site

Age	LD	MH	DT	HZ	GL
**2**	17.76 ± 1.04 c	10.84 ± 0.93 b	7.05 ± 0.51 b	3.64 ± 0.28 c	0.75 ± 0.13 c
**3**	57.97 ± 3.91 bc	36.48 ± 1.83 b	56.11 ± 4.01 a	22.94 ± 1.83 bc	11.97 ± 1.38 bc
**4**	91.05 ± 5.54 ab	75.98 ± 5.71 a	64.86 ± 4.03 a	31.54 ± 1.48 ab	26.05 ± 1.96 ab
**5**	102.01 ± 7.14 a	78.87 ± 4.01 a	62.44 ± 3.33 a	42.84 ± 2.16 a	41.69 ± 2.14 a

Note: Values in the same column followed by the same letter are not significantly different. The significance level was set to *P* < 0.05. Field sampling sites: LD (Ledu); MH (Minhe); DT (Datong); HZ (Huzhu); GL (Guoluo)

**Fig. 1:**
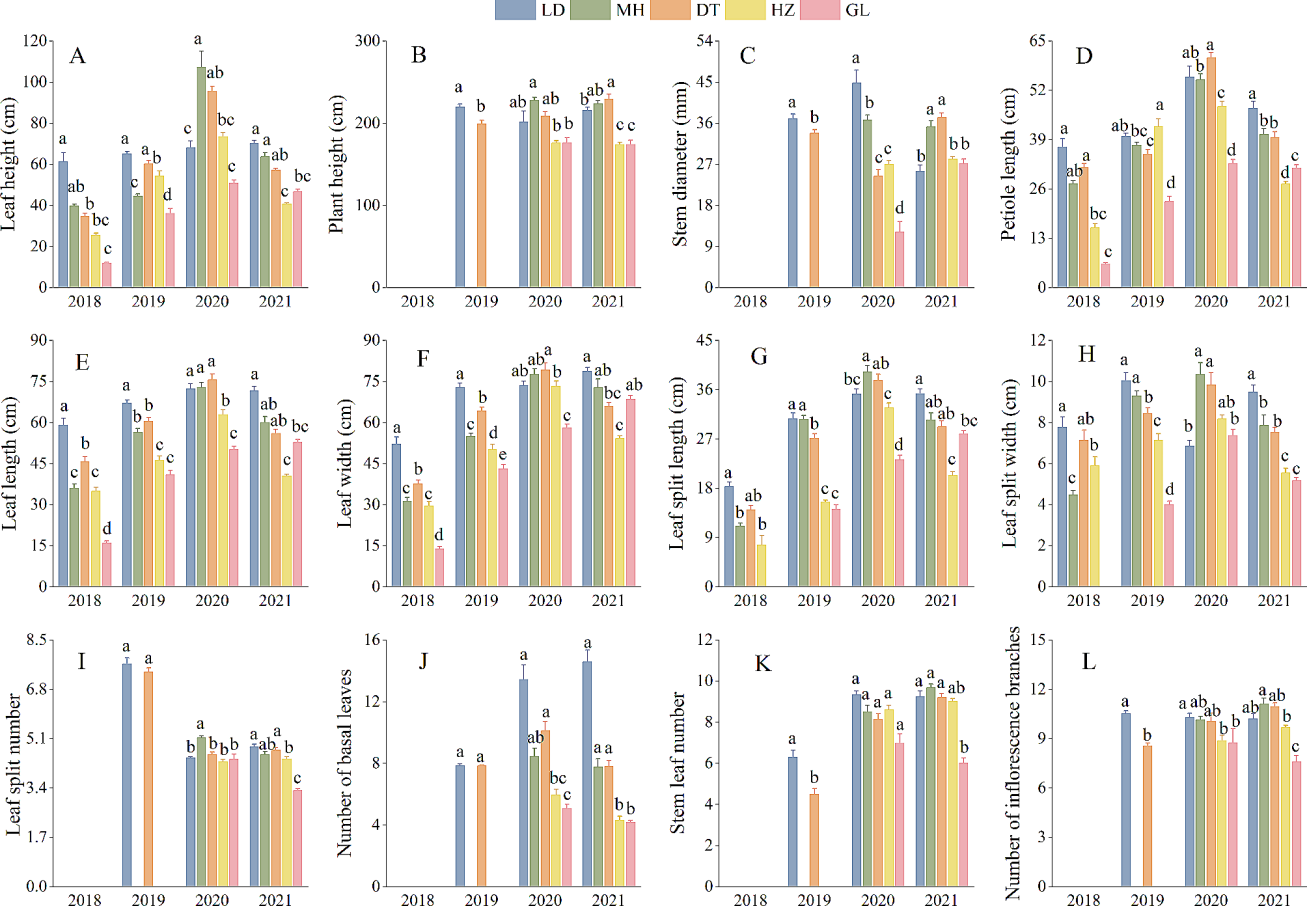
Comparison of aboveground traits of same-aged plants grown at different experimental sites (LD: Ledu; MH: Minhe; DT: Datong; HZ: Huzhu; GL: Guoluo). Significant differences at *P* < 0.05 are indicated by different letters

Profiles of the above-ground characteristics of *R. tanguticum* across different altitudes are summarized in Fig. 1. In summary, plants growing at low altitudes displayed better characteristics than their high-altitude counterparts. Notably, samples from the GL and Huzhu (HZ) experimental sites had significantly lower growth performance values than those from the LD, Minhe (MH) and DT sites. Five-year-old plants had a height of up to 229.5 cm, and their number of stem leaves and inflorescence branches also reached a maximum (Fig. 1B, K and L). Four-year-old plants displayed the highest stem diameter (44.6 mm), petiole length (60.5 cm), leaf length (75.6 cm), leaf width (79.2 cm), leaf split length (39.2 cm), leaf split width (10.3 cm) and leaf split number (5.1), although these values were not significantly different from those of 5-year-old plants (Fig. 1 C- 1I). Four-year-old plants in LD had significantly higher stem diameters than those from the other four sites, and those in GL were the lowest (12.16 mm) (Fig. 1 C). The stem diameter of 5-year-old plants from DT was significantly higher than that of LD, HZ and GL, although there was no statistically significant difference between MH and DT. Four-year-old plants had the longest petiole length (60.54 cm) at the DT experimental site (Fig. 1D). We found no significant differences in longest petiole length in LD, but the values were significantly higher than those in MH, HZ and GL. Five-year-old plants from the LD site had the maximum petiole length (47.2 cm), which was significantly higher than that of the other sites. However, we found no significant differences in petiole length between MH and DT.

**Fig. 2:**
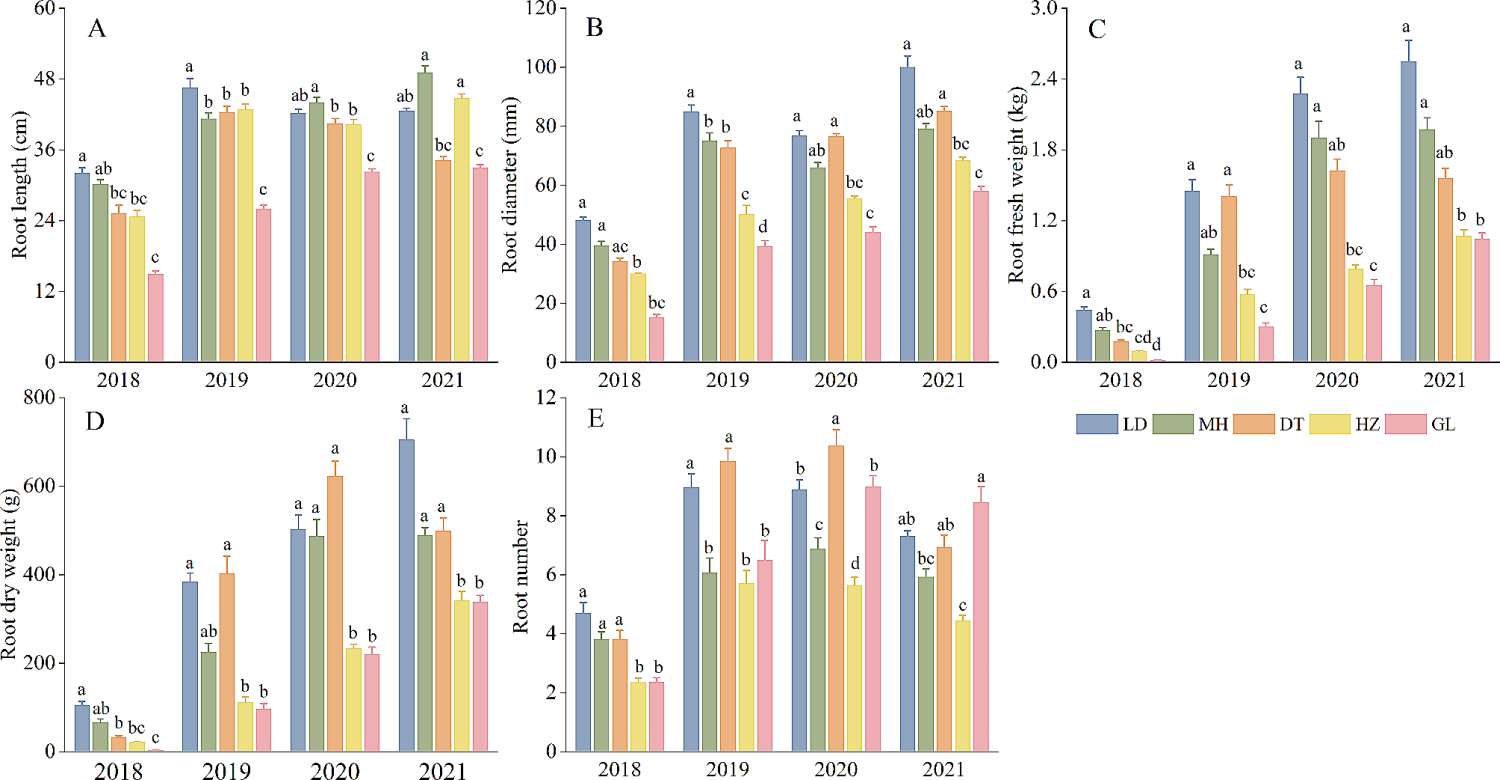
Comparison of underground traits among same-aged plants grown in different experimental sites (LD: Ledu; MH: Minhe; DT: Datong; HZ: Huzhu; GL: Guoluo). Significant differences at *P* < 0.05 are indicated by different letters

Profiles of underground traits, namely, root length, root diameter, root fresh weight, root dry weight, and root number of plants across different altitudinal gradients, are shown in Fig. 2. In summary, the root trait value decreased with increasing altitude for the 2-year-old sample. The LD experimental site had the highest root diameter and root fresh weight (40.14 mm and 0.44 kg), while GL had the lowest(15.12 mm and 0.02 kg) (Fig. 2B C). Plants growing in LD and DT had the highest root dry weight, while GL had the lowest. Notably, 3-, 4-, and 5-year-old samples from low-altitude areas (LD, MH, and DT) had better performance across the aforementioned 3 parameters than those from high-altitude sites (HZ, GL). Root number displayed a W-shaped trend in the altitude gradient from 3 to 5 years old, as evidenced by relatively high LD, DT and GL values and relatively low MH and HZ. In addition, 3- and 5-year-old plants at the GL site had significantly lower root lengths than those at the other experimental sites.

### **Effect of altitudinal gradients on** ***R. tanguticum*constituents**

Interannual comparisons of total anthraquinone content for *R. tanguticum* are shown in Table 2. In summary, the total anthraquinone contents exhibited a consistent trend across the five experimental sites and increased with increasing plant age. The total anthraquinone content of 2-year-old plants was significantly lower than that of 4- and 5-year-old plants. We also found no statistically significant differences in total anthraquinone content between 4- and 5-year-old plants across LD, DT and HZ. Moreover, the total anthraquinone content of 3-year-old roots reached the pharmacopoeia standard (2020 edition) at all sites and showed continued accumulation during later stages of plant growth. Notably, the content in 5-year-old samples was 2–3 times higher than that in the pharmacopoeia standard (2020 edition). In summary, DT had the highest contents (5.15%), followed by MH and LD, while GL had the lowest (3.52%).

**Table 2:** Interannual comparison of total anthraquinone in different experimental sites (%/plant)

Ages	LD	MH	DT	HZ	GL
**2**	1.99 ± 0.12b	1.53 ± 0.10d	1.72 ± 0.09c	1.1 ± 0.04c	0.82 ± 0.07c
**3**	3.28 ± 0.34ab	2.5 ± 0.15c	3.29 ± 0.26bc	2.47 ± 0.16bc	2.01 ± 0.22b
**4**	4.06 ± 0.21a	3.29 ± 0.13b	4.39 ± 0.14ab	2.85 ± 0.09ab	2.39 ± 0.11b
**5**	4.19 ± 0.14a	4.26 ± 0.24a	5.15 ± 0.15a	3.97 ± 0.21a	3.52 ± 0.15a

Note: Values in the same column followed by the same letter are not significantly different. The significance level was set to *P* < 0.05. Field sampling sites: LD (Ledu); MH (Minhe); DT (Datong); HZ (Huzhu); GL (Guoluo)

**Fig. 3:**
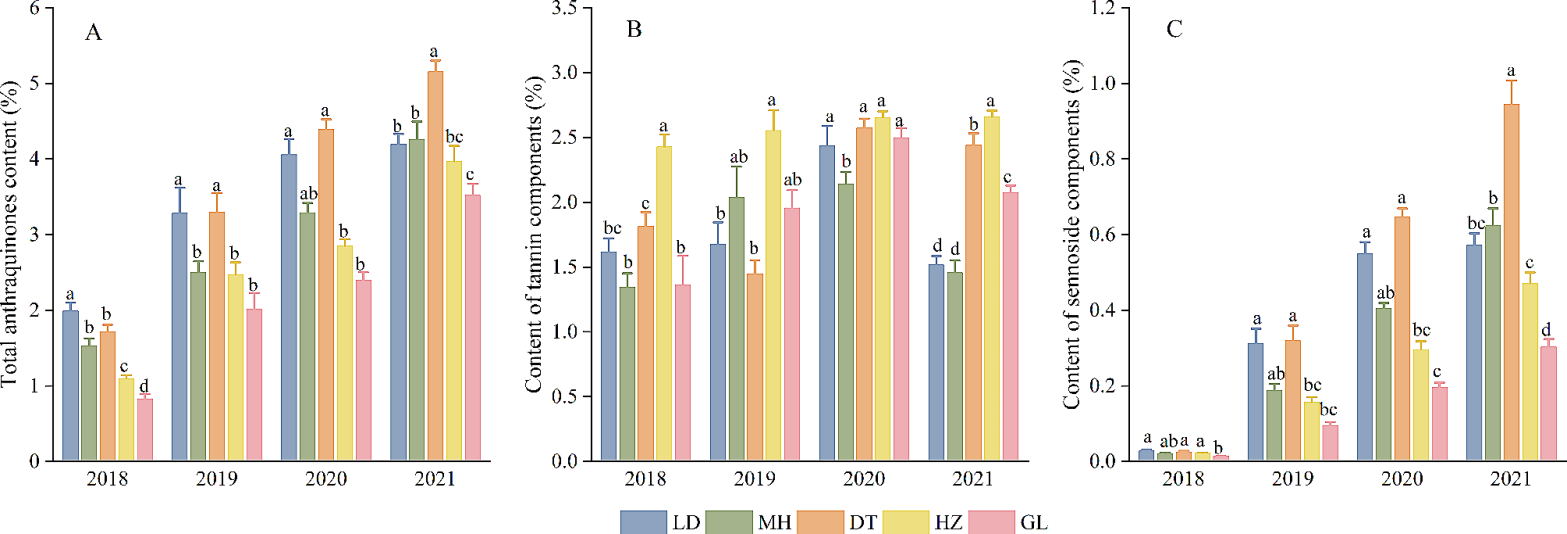
Comparison of differences in the active constituents of *R. tanguticum* at different experimental sites (LD: Ledu; MH: Minhe; DT: Datong; HZ: Huzhu; GL: Guoluo). Significant differences at *P* < 0.05 are indicated by different letters

To further understand the effect of altitude on active ingredients, we compared total anthraquinones, total tannins and total sennoside of plants across the five experimental sites with those in one year. The results are shown in Fig. 3. In summary, the highest contents of all three metabolites were recorded at low- and middle-altitude sites. The highest total anthraquinone content was recorded in plants grown in the LD and DT sites, while the lowest was observed in the HZ and GL sites (Fig. 3A). When the altitude exceeded a certain range, the content decreased with increasing altitude. On the other hand, the highest total tannin content was recorded in HZ (2.66%) (Fig. 3B), while the highest total sennoside content was observed in DT (0.94%), which decreased with increasing altitude (Fig. 3C). The content was the lowest at the GL site (0.30%).

### **Metabolite profiles of** ***R. tanguticum*** **grown at different altitudes**

**Fig. 4:**
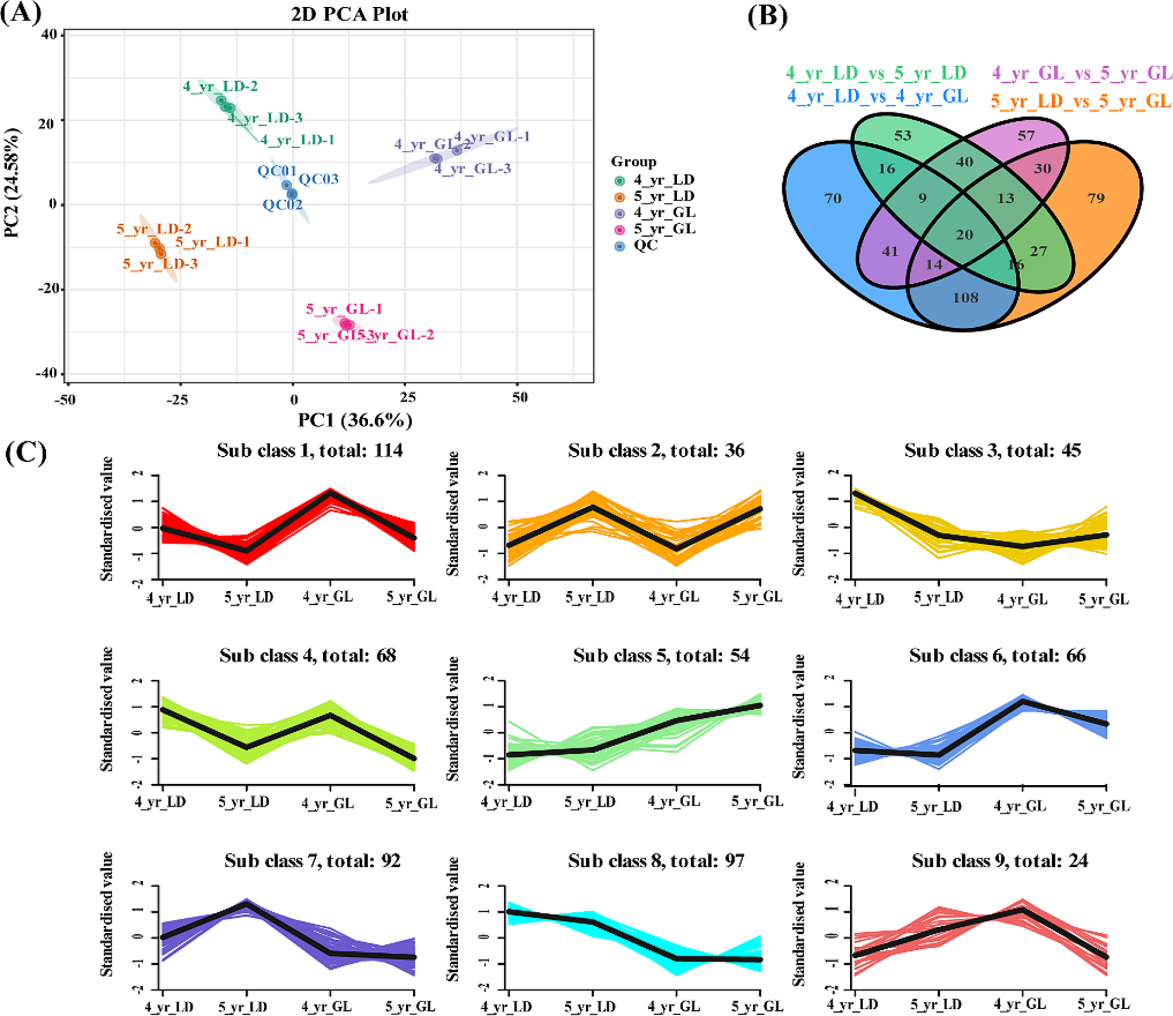
Differentially abundant metabolite profiles in total samples. (**A**) PCA showing sample clustering; (**B**) Venn diagram; (**C**) K-means clustering trend graph

To assess whether altitudinal gradients affect the secondary metabolites of *R. tanguticum*, we employed wide-target metabolomics to analyze differences in root samples of plants grown at two experimental sites (LD and GL). The UPLC‒MS/MS analysis platform and MetWare self-built database were used to detect a total of 1400 metabolites in root samples, which were mainly divided into the following categories: alkaloids (8.07%), amino acids and their derivatives (8.71%), flavonoids (18.36%), lignin and coumarins (3.36%), lipids (10.07%), nucleotides and their derivatives (4.36%), organic acids (5.57%), phenolic acids (19.07%), quinones (5.14%), tannins (1.29%), terpenoids (3.57%) and others (12.43%). We performed principal component analysis (PCA) and found that the QC samples were located in the middle of the four groups, which closely clustered together (Fig. 4). Replicates of each sample were also clustered together, indicating that the analysis had good stability and reproducibility. In the PCA, the first two principal components accounted for 61.18% (Fig. 4A). PC1 explained 36.60%, and the first principal component could distinguish samples from different locations. PC2 explained 24.58%, and the second principal component showed differences between years. Metabolites could be used to clearly distinguish between samples from different years and sites, indicating that the secondary metabolites of *R. tanguticum* were different under altitude conditions. We generated a Venn diagram to depict the differential distribution of these metabolites across different groups (Fig. 4B). In summary, we detected a total of 593 differentially abundant metabolites across the four groups, of which 294 and 307 were differentially expressed in 4- and 5-year-old plants, respectively, in LD and GL. A total of 158 differentially abundant metabolites between the two groups indicated that they all responded to altitude stress in the two-year experiment.

Next, we used K-means clustering to cluster the differentially abundant metabolites into 9 categories (Fig. 4C). Here, 3 subclasses displayed a consistent trend in changes between the two years. With increasing altitude, Sub class 5 showed a relative content increase, while the relative contents of Sub class 4 and Sub class 8 decreased. Flavonoids, alkaloids and phenolic acids were the most abundant metabolites in Subclasses 5, 4, and 8, respectively. Notably, tannins were found in Sub class 5 but not in the other two categories.

**Fig. 5:**
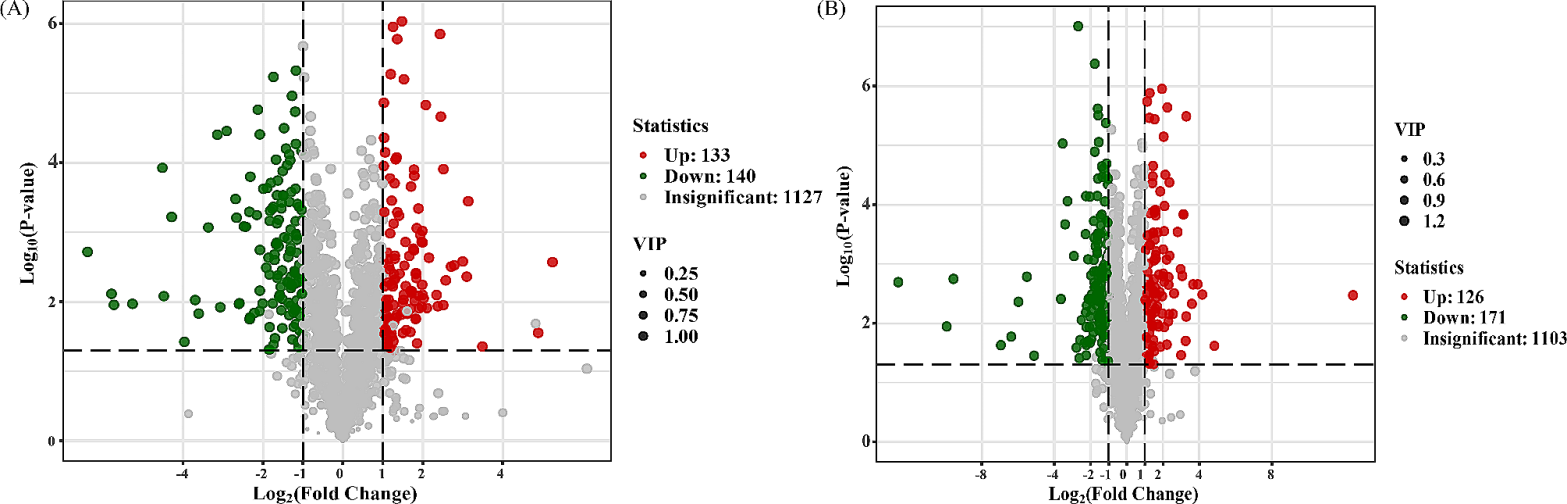
Expression profiles of R. tanguticum metabolites at different altitudes. **A**: 4-year-old plants; **B**: 5-year-old plants. Red and green dots denote significantly upregulated and downregulated metabolites, respectively, while gray dots indicate nonsignificantly expressed metabolites

A volcano plot of 4-year-old samples showed that 140 metabolites were significantly downregulated in the upper left area, while 133 were significantly upregulated in the upper right area (Fig. 5A). In 5-year-old *R. tanguticum* plants, 126 and 171 differentially abundant metabolites were significantly upregulated and downregulated, respectively (Fig. 5B). The significantly upregulated metabolites in response to altitude stress across the two years included flavonoids, which was consistent with the results of k-means clustering. Conversely, phenolic acids and lipids were significantly downregulated.

**Fig. 6:**
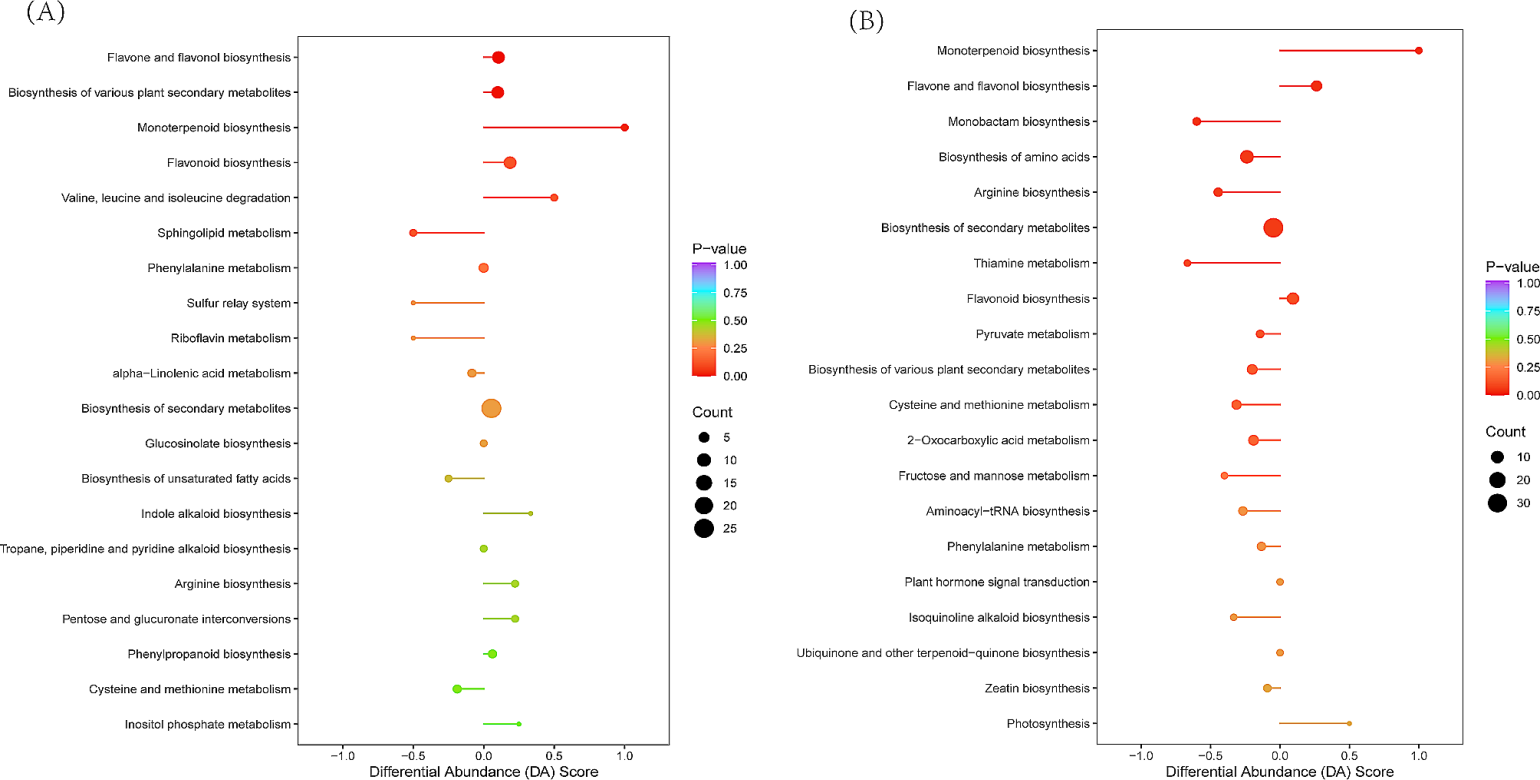
KEGG pathway results showing enriched pathways (**A**: 4-year-old plants; **B**: 5-year-old plants)

**Fig. 7:**
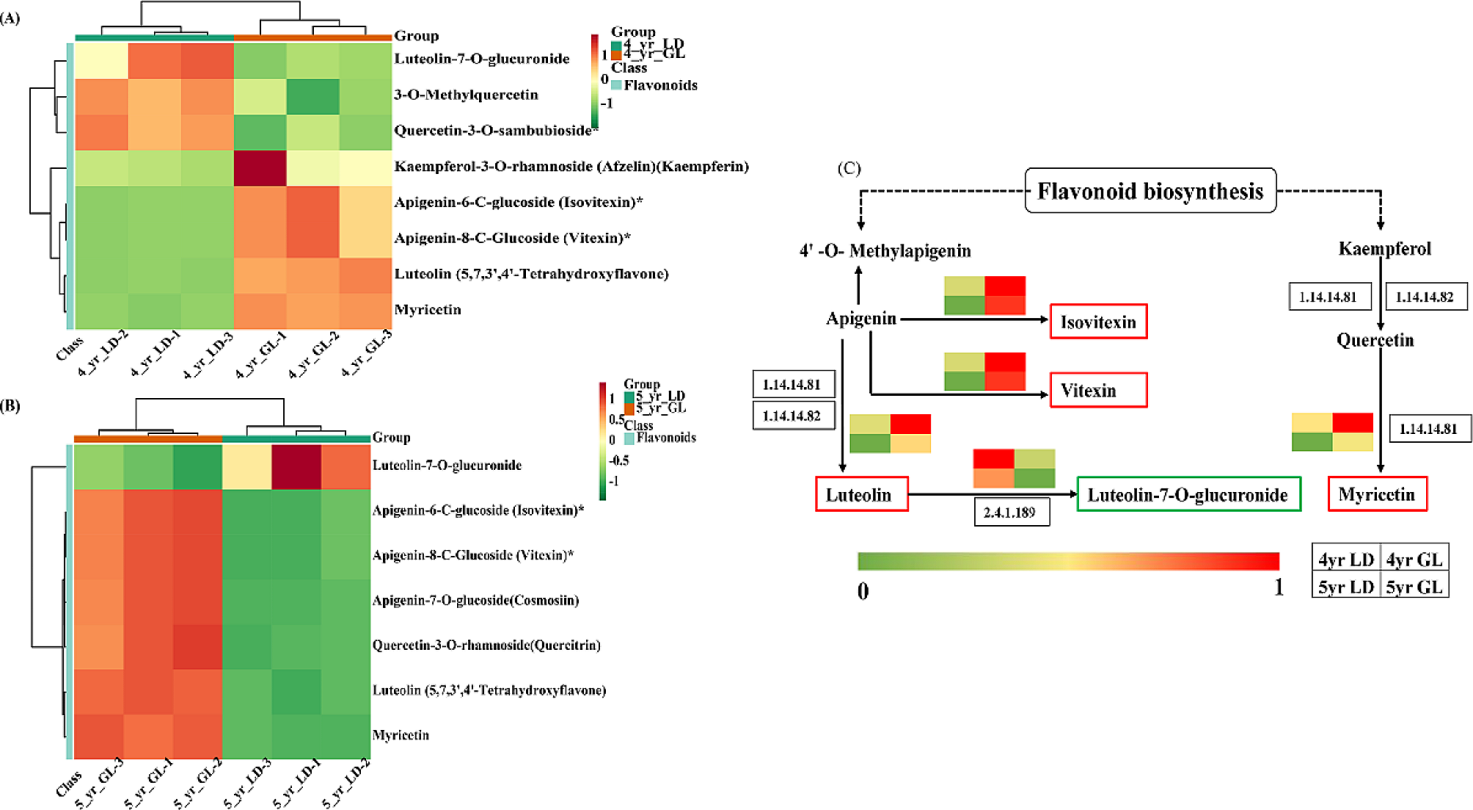
KEGG cluster heat map and metabolic network map (**A**: 4-year-old plants; **B**: 5-year-old plants; C: The expression of metabolites with altitude differences shared across different years in the ko00944 pathway.)

Next, we subjected the identified metabolites to Kyoto Encyclopedia of Genes and Genomes (KEGG) analysis to identify enriched pathways and found enrichment across three main categories, namely, metabolism, genetic information processing and environmental information processing. The larger and redder the dot, the more important it is for the signaling pathway. Figure 6 shows that metabolite pathways differentially enriched at different altitudes in the two-year experiment contained flavonoid and flavonol biosynthesis pathways and monoterpene biosynthesis pathways. The abundance score map showed that these two pathways were distributed on the right side of the central axis, and the line segment was longer, indicating that these metabolites were upregulated. The former enriched 7–8 differentially abundant metabolites (8 in 4-year-old samples of Figs. 6A and 7 in 5-year-old samples of Fig. 6B), and the latter was 2. These results indicated that the flavonoid and flavonol biosynthetic pathways played an important role in the plant response to altitude stress.

A cluster heatmap of differentially abundant metabolites in the KEGG pathway showed that luteolin, isovitexin, vitexin and myricetin were negatively and positively correlated with LD and GL roots, respectively, while luteolin-7-O-glucuronide exhibited the opposite trend. Different altitudes were associated with different contents of secondary metabolites (Fig. 7A and B). The differentially expressed metabolites between years exhibited considerable overlap, which indicated the similarity in the anti-stress mechanism of rhubarb under different altitude conditions (Fig. 7). Next, we normalized the relative content of the five common components and then generated a pathway heatmap in combination with the biosynthesis of flavonoids and flavonols (ko00944) pathway. Notably, high altitude was associated with an increase in the content of flavonoids, such as luteolin, isovitexin, vitexin and myricetin, while that of luteolin-7-O-glucuronide decreased (Fig. 7C). Five overlapping differentially abundant metabolites were considered the key metabolites in response to stress.

### Assessment of the response of the quality to environmental factors based on redundancy analysis

**Fig. 8:**
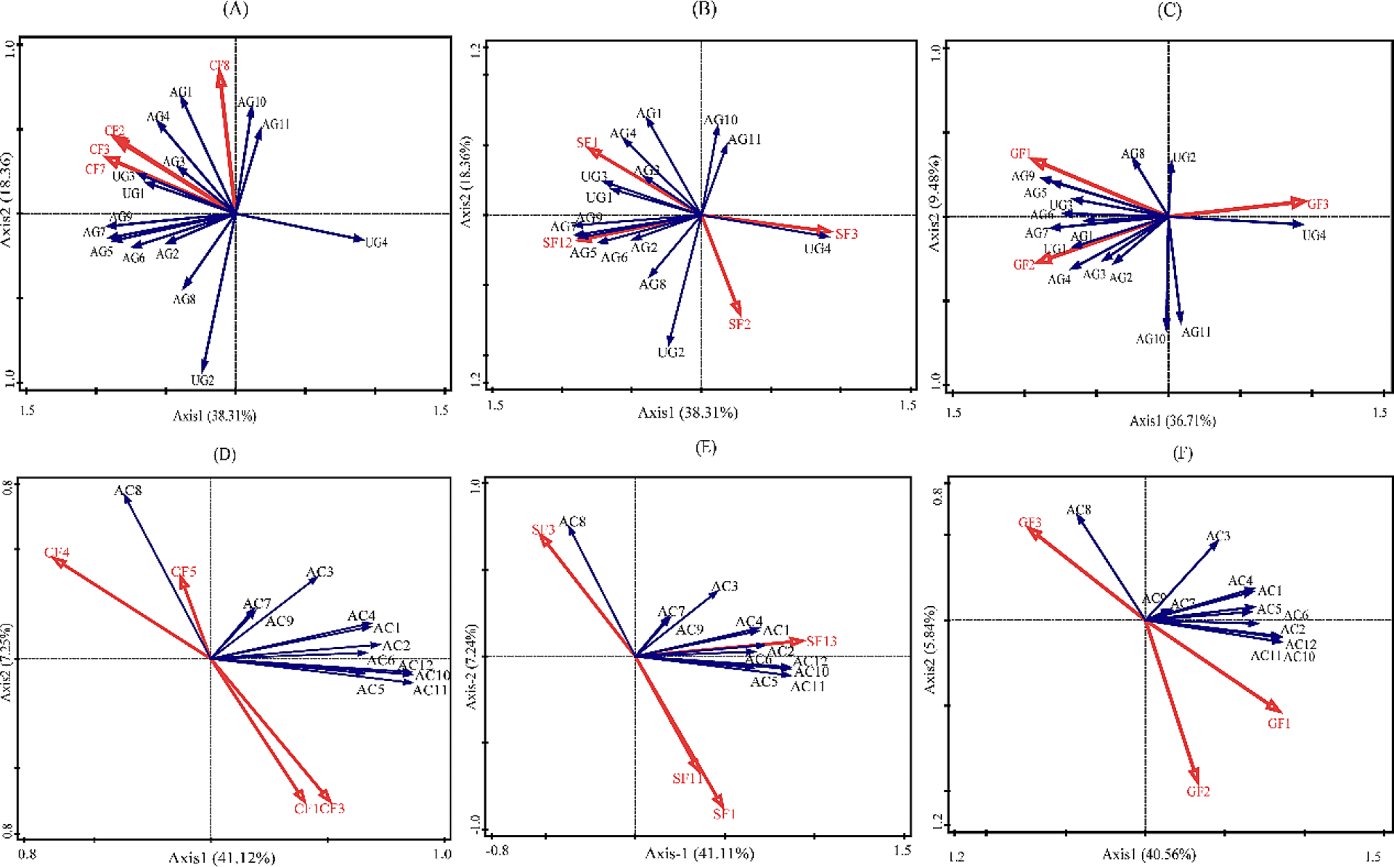
Redundancy analysis of the effect of ecological factors on active ingredients and growth traits (**A-C**: Redundancy analysis of the growth traits with climate factors, soil factors, and geographical factors, respectively; **D-F**: Redundancy analysis of the active ingredients with climate factors, soil factors, and geographical factors, respectively; AG1: number of basal leaves; AG2: leaf height; AG3: plant height; AG4: stem thickness; AG5: leaf length; AG6: leaf width; AG7: leaf split length; AG8: leaf split width; AG9: petiole length; AG10: number of stem leaves; AG11: number of inflorescence branches; UG1: root length; UG2: root diameter; UG3: fresh root weight; UG4: dry root weight; AC1: total anthraquinones; AC2: Aloe emodin; AC3: rhein; AC4: emodin; AC5: chrysophanol; AC6: emodin methyl ether; AC7: total tannins; AC8: gallic acid; AC9: catechins; AC10: total sennosides; AC11: sennoside B; AC12: sennoside A; CF1: annual average temperature; CF2: annual maximum temperature; CF3: annual minimum temperature; CF4: annual rainfall; CF5: average relative humidity; CF6: average water vapor pressure; CF7: average atmospheric pressure; CF8: annual sunshine hours. GF1: Latitude; GF2: longitude; GF3: altitude; SF1: pH; SF2: organic carbon; SF3: soil bulk density; SF4: clay content; SF5: sand content soil; SF6: cation exchange capacity; SF7: conductivity; SF8: 0–10 cm total nitrogen; SF9: 10–20 cm total nitrogen; SF10: 20–30 cm total nitrogen; SF11: 0–10 cm available phosphorus; SF12: 10–20 cm available phosphorus; SF13: 20–30 cm available phosphorus; SF14: 0–10 cm organic matter; SF15: 10–20 cm organic matter; SF16: 20–30 cm organic matter)

The index of active ingredients and the growth trait index were used as response variables, while climatic, soil, and geographical factors were used as explanatory variables for redundancy analysis. The data were standardized using the ‘Center and Standardization’ method, and environmental variables were analyzed using the ‘Forward Selection’ method. The results of the six models are shown in Fig. 8. After the replacement test, the selected variables and models were significant (*P* < 0.05).

The corrected correlation coefficient R^2^ of climatic factors in the redundancy analysis model of active ingredients was 0.479, and climatic factors, including annual rainfall, annual minimum temperature, annual average temperature, and average relative humidity, had the greatest impact. The annual average and minimum temperature were negatively correlated with gallic acid but positively correlated with anthraquinones and sennosides. On the other hand, the annual rainfall had an opposite effect on the level of active ingredients (Fig. 8D). The correction coefficient (R^2^) for the relationship between climatic factors and growth indicators was 0.615. Climatic factors, including annual sunshine hours, annual maximum temperature, annual minimum temperature, and average pressure, had the greatest impact on growth. These indicators have positive effects on most aboveground and underground growth. The results showed that warm and rainless conditions mostly favored the growth of *Rheum tanguticum* (Fig. 8A).

The correction coefficient for the association between soil factors and the secretion of active ingredients was 0.473, and soil factors, including pH, 0–10 cm available phosphorus, and 20–30 cm available phosphorus, had the greatest effect on the content of active ingredients. Soil pH and available phosphorus between 0 and 10 cm depths were negatively correlated with gallic acid content, while available phosphorus at 20–30 cm depth was positively correlated with catechin, anthraquinone, and sennoside contents (Fig. 8E). The R^2^ of the growth index redundancy analysis model was 0.452, and the soil factors, including pH, 10–20 cm available phosphorus, soil bulk density, and organic carbon, significantly affected it. Soil pH and 10–20 cm available phosphorus were positively correlated with leaf traits and root traits. On the other hand, organic carbon and soil bulk density were negatively correlated with leaf traits and root traits (Fig. 8B). The results showed that an alkaline soil environment was suitable for cultivating rhubarb.

The correction R^2^ of geographical factors in the redundancy analysis model of active ingredients was 0.452, and important geographical factors, including longitude, latitude, and altitude, affected it. Altitude was positively correlated with gallic acid content and negatively correlated with the content of other active ingredients. Latitude was strongly and negatively correlated with the gallic acid content, whereas latitude was positively correlated with other active ingredient contents (Fig. 8F). The correction R^2^ of the growth index redundancy analysis model was 0.456, and the important geographical factors were longitude, latitude, and altitude. Latitude was positively correlated with root diameter and fresh root weight. Moreover, longitude was positively correlated with aboveground traits. Altitude was positively correlated with root dry weight and negatively correlated with other growth traits. The latitude and longitude variations have a significant impact on plant composition and growth (Fig. 8C). Although the latitude and longitude differences (in “second”) in our experimental sites are not at a high level, there are substantial variations in soil pH, precipitation, and other factors among the sites, which may potentially influence the research results. Therefore, future studies need to pay more attention to and address the latitude and longitude differences, and conduct relevant latitude and longitude gradient research to gain a deeper understanding of the mechanisms by which latitude and longitude variations affect the medicinal herb quality.

### Redundancy analysis of differentially abundant metabolites and ecological factors

**Fig. 9:**
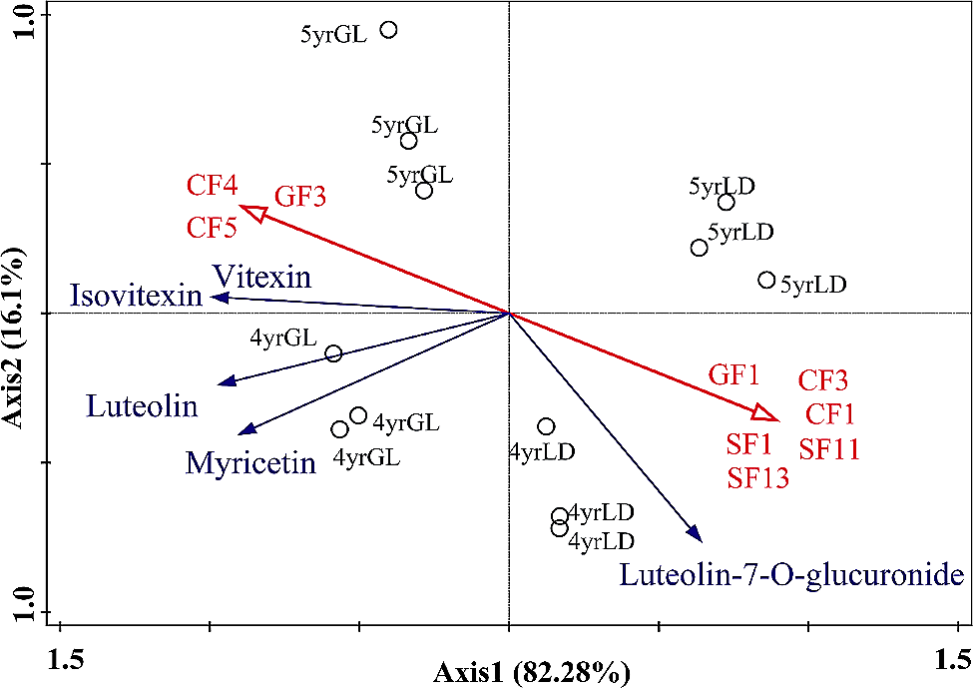
Redundancy analysis of differential metabolites and ecological factors (4yrGL: 4-year-old plants in Guoluo; 4yrHZ: 4-year-old plants in Huzhu; 5yrGL: 5-year-old plants in Guoluo; 5yrHZ: 5-year-old plants in Huzhu; CF1: annual average temperature; CF3: annual minimum temperature; CF4: annual rainfall; CF5: average relative humidity; GF1: Latitude; GF3: altitude; SF1: pH; SF11: 0–10 cm available phosphorus; SF13: 20–30 cm available phosphorus)

The ecological factors identified using the previous redundancy analysis model and the differentially abundant metabolites present in the two-year trials based on the KO00944 pathway were subjected to redundancy analysis (Fig. 9). Samples collected from the same site and year in the two-year trial were highly similar. Annual rainfall, altitude, and average relative humidity were strongly and positively correlated with luteolin, isovitexin, vitexin, and myricetin content. Moreover, the relative content of these four components increased with altitude. Luteolin-7-O-glucuronide is a downregulated component in the pathway, and its content is positively correlated with latitude, annual minimum temperature, pH, annual average temperature, 0–10 cm available phosphorus, and 20–30 cm available phosphorus. In addition, the relative content of luteolin-7-O-glucuronide was also downregulated. The results showed that environmental factors significantly affected the content of the five components at GL. The effect of ecological factors on flavonoids further affected quality. These compounds played an important role in the synthesis of active substances and antioxidant activity.

## Discussion

The gap between the growing demand for Chinese medicinal materials and the supply of medicinal resources has been one of the bottlenecks in the development of the Chinese medicine industry [19]. Developing and planting high-quality cultivars may be the most efficient approach to solve this dilemma. However, studies investigating the ecological requirements of *R. Tanguticum* are scarce [20]. Plant growth traits play a significant role in determining the yield and quality of herbs. Therefore, understanding changes in different localities and years could reveal the inherent trends in plant growth and quality [21]. Habitat is closely related to plant size, number, and resource allocation [22]. Previous research has shown that growth parameters, including height, leaf length, and leaf width growth, decrease with altitude [23]. For example, the rhizome of *Coptis chinensis* Franch is longer at low altitudes (2300 m) than at high altitudes (2600 m, 2700 m) [24], consistent with our findings. Root biomass decreases with an increase in altitude. Additionally, roots are morphologically very diverse. The roots in plants grown in GL were small and had many lateral roots, whereas the roots in HZ were larger, with smooth and clear differentiation between main and lateral roots. Second, the aboveground parts of *R. tanguticum* in low-altitude areas varied from those in high altitudes. Altitude affects the phenological period of plants [25]. We found that later growth and development were higher at higher altitudes than at lower altitudes. This explains the missing data for plant height and stem thickness at HZ and GL in 3-year-old plants. Overall, in our research, aboveground growth was better at low altitudes than at high altitudes. Previous studies have shown that high UV rays may be a major contributor to harsh environmental factors at high altitudes [26–27]. The small size of *R. tanguticum* leaves at higher altitudes could be attributed to high UV radiation, which may have influenced transpiration-driven water loss [28].

The quality of Chinese medicinal materials is affected by cultivation methods and environmental conditions, resulting in different syntheses and the accumulation of secondary metabolites. Different types and contents of secondary metabolites are responsible for the varying quality of Chinese medicinal materials [29]. Our results showed that the lowest anthraquinone content was at the GL site, and the highest content was at the LD or DT site in different years. However, the DT experimental site did not conform to this pattern, where the content decreased with increasing altitude, which could be attributed to soil fertility and the previous crop. The decomposition products negatively or positively affect the subsequent crops. For example, the total alkaloid content of *Ephedra saxatilis* was found to be higher than that of *Ephedra gerardiana* with an increase in soil moisture and temperature. Conversely, when the soil moisture was lower, both *Ephedra* species exhibited higher alkaloid content. Therefore, it can be concluded that high altitude combined with water deficit conditions may be more favorable for *Ephedra* in producing higher amounts of alkaloids [30]. Moreover, our study revealed that the contents of sennoside components decreased with increasing altitude. The tannin content was highest at HZ across seasons, suggesting that the content was higher at high altitudes and lower at middle and low altitudes, consistent with previous studies on rhubarb. Yan et al. also showed that the contents of anthraquinone and tannins were highest at 1400–1700 m [31]. This result was contrary to our findings, and the reason may be that the altitude range in the previous study was below our lowest altitude, which possibly limited the increase in active ingredients beyond a certain altitude. Similar findings have been reported in *Gentiana cras-sicaulis* Duthie ex Burk. The four ingredients followed a bell-shaped distribution within the altitudinal range of 2264–3100 m [32]. The anthraquinone, total tannin, and total sennoside contents increased with age and peaked at 4 or 5 years old, after which no significant difference was observed between these two ages. The results of other studies showed that the best harvest period for *R. tanguticum* based on the content of the main functional component was over four years [16, 33].

Plants alter their tissue structure and chemical composition to regulate their responses to external stimuli. Our study validated the influence of environmental factors on plant growth characteristics and ingredient content via redundancy analysis (RDA). An increase in sunshine days and the average annual temperature improved the growth of *R. tanguticum*. Rainfall was negatively correlated with the content of effective components, consistent with a previous study [20]. These results showed that the region with a high level of sunshine and low precipitation was conducive to the formation and accumulation of anthraquinone. Soil is the basic material for plant growth and development and for nutrient acquisition. Soil pH and 10–20 cm available P promoted leaf and root growth, while organic carbon and soil bulk density had the opposite effect. Our results show that *R. tanguticum* planted at low altitudes has higher quality and yield. The chemical characteristics of soil microenvironments, such as pH value and organic matter content, can influence nutrient availability and soil enzyme activity. The microbial communities in soil microenvironments play a crucial role in organic matter decomposition and nutrient cycling. These factors cannot be compensated for by a gridded dataset. Therefore, it is necessary for us to conduct further research in order to uncover the characteristics, regulatory mechanisms, and ecological functions of soil microenvironments in the future.

Along various altitudes, environmental conditions vary significantly, which might influence plant performance and distribution [34]. As a result of adaptive plant strategies, plants regulate their growth and development in response to abiotic stresses through primary and secondary metabolism [35]. The metabolomics results showed that *R. tanguticum* contains phenolic acids, flavonoids, alkaloids, quinones, and lipids, which have antibacterial, anti-inflammatory, anticancer, and immunomodulatory properties [36, 37]. Along the altitudinal gradient, differentially abundant metabolites were enriched in the biosynthesis of flavonoids and flavonols and the monoterpenoid biosynthesis pathway. The number of differentially abundant metabolites was higher in the former and most of which were abundant in the GL experimental site, which may have an important effect on the quality of the plants. The high content of lipids and phenolic acids in LD endowed *R. tanguticum* at lower altitudes with a unique quality. Metabolites were linked to climatic and other factors [38]. Altitude, annual rainfall, and average relative humidity were higher in GL, and these environmental factors affected isovitexin, vitexin, myricetin, and luteolin production. These metabolites have antioxidant, hypoglycemic, anti-inflammatory, and other pharmacological effects [39]. However, annual rainfall and average relative humidity were negatively correlated with anthraquinone contents. Therefore, it can be inferred that anthraquinones dominating high-altitude areas have lower laxative qualities but higher antioxidant qualities. Consistent with our results, the flavonoid content of *Agriophyllum squarrosum* was strongly and positively correlated with precipitation. On the other hand, the flavonoids of *Agriophyllum squarrosum* were negatively correlated with temperature, suggesting that the accumulation of flavonoids may result from adaptation to environmental heterogeneity [40].

## Conclusion

**Fig. 10:**
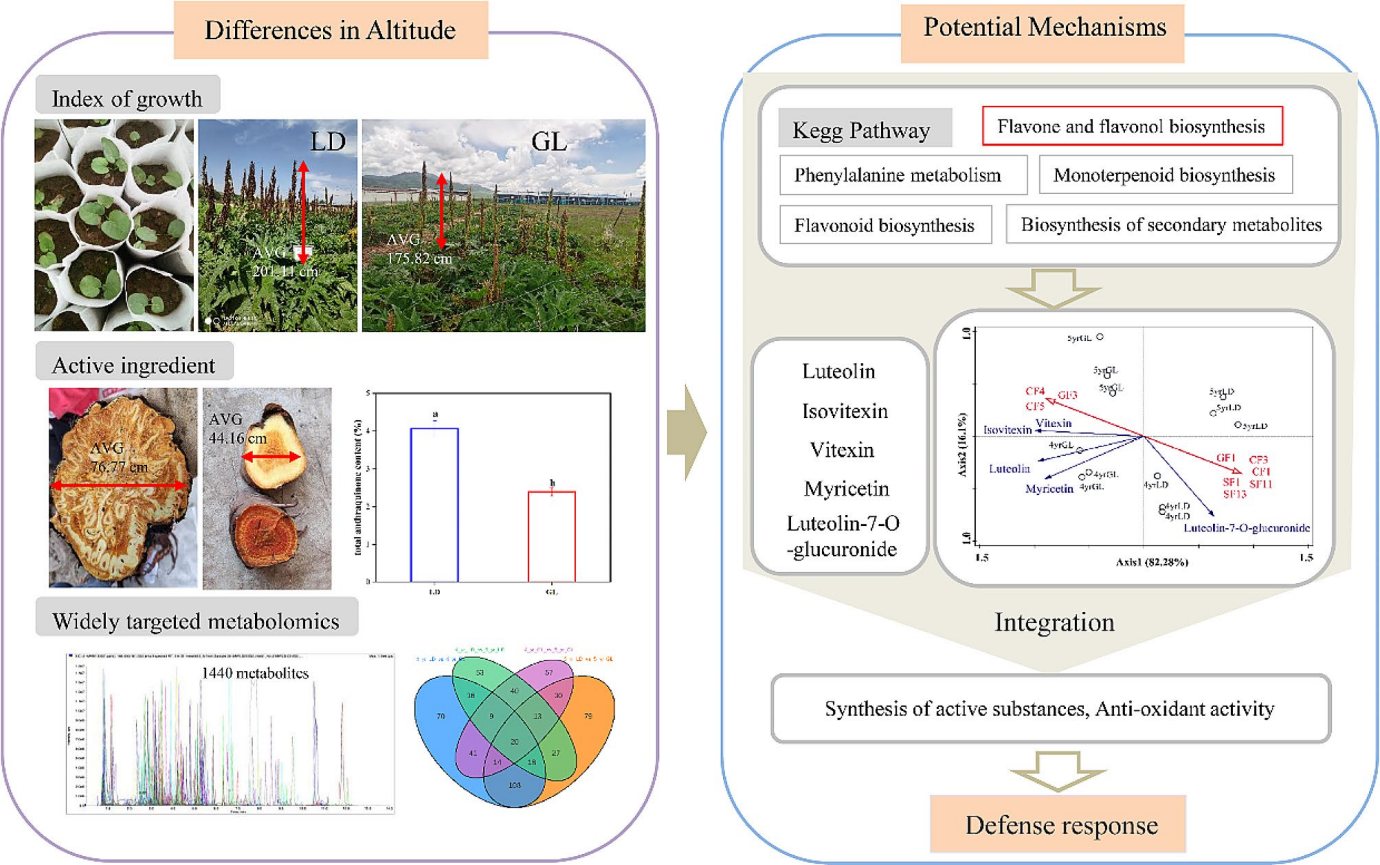
Adaptation mechanism to altitude diagram

The present study analyzed the growth traits and active ingredients of *R. tanguticum* at different ages at the five altitudinal gradient sites via a widely targeted metabolomics and RDA approach. Our data indicated that above- and underground growth traits showed better properties in low-altitude sites. Additionally, extracts from *R. tanguticum* showed variations among sites and plant ages. Redundancy analyses revealed that the variations in *R. tanguticum* might be related to geographical location (latitude, longitude, and altitude) and environmental variables (climate and soil). Among them, annual precipitation, annual average temperature, and soil pH were prominent factors influencing differences in active ingredient contents and growth traits in *R. tanguticum*. The most critical reason for the high content of anthraquinones in low-latitude sites may be the low precipitation and slightly basic soil pH. This was also the reason why flavonoid contents were higher at high-altitude sites than at low-altitude sites (Fig. 10). Furthermore, our study results are significant for informing site selection and the systematic cultivation of *R. tanguticum*.

## Methods

### Plant materials

*R. tanguticum* plants, at four ages, were collected from five experimental sites, namely, Ledu (LD, 2016 m), Minhe (MH, 2180 m), Datong (DT, 2409 m), Huzhu (HZ, 2971 m), and Guoluo (GL, 3763 m), in the Qinghai–Tibet Plateau region in eastern Qinghai Province, China. The age of plants used in this study ranged from 2 to 5 years as follows: 2, 3, 4 and 5 years in 2018, 2019, 2020, and 2021, respectively. The samples were identified as *R. tanguticum* by Prof. Guoying Zhou, Northwest Institute of Plateau Biology, Chinese Academy of Science. The voucher specimens and their information were deposited at the Qinghai-Tibetan Plateau Museum of Biology, Chinese Academy of Science (QHGC-1813). In each sample, we measured 11 aboveground traits, including leaf and plant height, stem thickness, leaf length and width, leaf split length and width, petiole length, number of basal leaves, number of stem leaves and number of inflorescence branches. We also recorded root parameters, including length, diameter, fresh weight, and number, and used them to calculate the underground growth of *R. tanguticum* grown at different altitudes and at different ages. After the plants were harvested from the fields, entire roots were washed and cut into slices, which were subsequently dried and crushed into fine powder for composition analyses.

### Active ingredient analysis

#### Sample extraction

All methods were carried out in accordance with relevant guidelines. Anthraquinone standards were purchased from the National Institutes for Food and Drug Control (Beijing, China). Anthraquinone mixed reference solutions for aloe-emodin, rhein, emodin, chrysophanol and physcion were separately prepared by dissolving the accurately weighed reference standards in methanol (≥ 99.9%) to yield concentrations of 0.0084 mg/mL, 0.08 mg/mL, 0.02 mg/mL, 0.14 mg/mL and 0.2 mg/mL, respectively. Tannin and sennoside standards were obtained from Chengdu Desite Biotechnology Co., Ltd. (Chengdu, China). Tannin-mixed reference solutions for gallic acid and catechins were separately prepared by dissolving accurately weighed reference standards in methanol (≥ 99.9%) to yield concentrations of 0.20 mg/mL and 0.26 mg/mL, respectively. Sennoside mixed reference solutions for sennoside A and sennoside B were separately prepared by dissolving accurate volumes of reference standards in methanol (≥ 99.9%) to yield concentrations of 0.28 mg/mL and 0.20 mg/mL, respectively.

To prepare the anthraquinone sample, 0.15 g was weighed and placed in a conical flask, then 25 mL of methanol (≥ 99.9%) was added, and the flask was weighed. The contents were refluxed for 1 h in a condensation reflux device at 65 °C. The mixture was cooled and weighed again, and the lost weight was made up with methanol (≥ 99.9%). Next, 5 mL of the solution was admixed with 10 mL 8% hydrochloric acid after drying at 65 °C using rotary evaporation and subjected to ultrasonication for 2 min. Then, 10 mL chloroform was added, and the sample was refluxed for 1 h. After cooling, the extract was transferred into a separating funnel, partitioned three times with chloroform (10 mL each time), and then spin-dried at 65 °C. The residue was dissolved in 10 mL methanol (≥ 99.9%) and then passed through a 0.22 μm membrane filter [41].

Extraction of tannins and sennosides and detection were performed according to our own laboratory method. Briefly, each sample was accurately weighed (0.5 g) and subsequently extracted with 25 mL 60% methanol (≥ 99.9%) for 1 h using ultrasonication (40 °C, 240 W, 40 kHz). After cooling, the contents were reweighed, and any lost weight was made up using 60% methanol (≥ 99.9%). Finally, the solution was passed through a filter membrane (0.22 μm) and stored at 4 °C until future use.

### Metabolite determination

Metabolite determination was achieved via high-performance liquid chromatography (HPLC, Agilent 1260, Agilent Technologies Co., Ltd., Shanghai, China) on a chromatograph equipped with a quaternary VL pump (G1311C), standard autosampler (G1329B), thermostatted column compartment (G1316A) and fluorescence detector (G1315D). The column was a C18 reversed-phase column (Agilent 5HC-C18, 250 × 4.6 mm), and the methanol (≥ 99.9%) and acetonitrile used in the mobiles phases were of HPLC grade and were procured from Shandong Yuwang Industrial Co., Ltd. (Shandong, China). The HPLC separation conditions for anthraquinone analysis were as follows: 0–25 min, liquid A linear gradient ranging from 42 to 45%; 25–55 min, liquid A linear gradient ranging from 45 to 80%. The column temperature was maintained at 25 °C at an analysis wavelength of 254 nm. The program for tannins and sennosides was as follows: 0–7 min, liquid A linear gradient ranging from 5 to 9%; 7–25 min, liquid A linear gradient ranging from 9 to 15%; 26–49 min, 15% liquid A; and 49–78 min, liquid A linear gradient ranging from 15 to 21%. Mobile phases A and B were acetonitrile and water containing 0.1% formic acid, respectively. The column temperature was 25 °C, and the analysis wavelength was 280 nm.

### Metabolomics data acquisition and analysis

Metabolomics is a research discipline that integrates the capabilities of several types of research fields, including analytical chemistry, statistics, and biochemistry [42]. Metabolomics assays were performed at Wuhan Maiteville Biotechnology Co., Ltd on 4- and 5-year-old *R. tanguticum* plants grown in the LD and GL regions. All reagents used, including acetonitrile, methanol ( ≥ ≥ 99.9%) and formic acid, were of chromatography grade. Data were acquired on an ultrahigh-performance liquid chromatography (UPLC) system coupled with tandem mass spectrometry (MS/MS). Mass spectrometry data were processed using Analyst 1.6.3 software, and the metabolites were qualitatively and quantitatively referenced against a local metabolic database [43]. Integration data for all chromatographic peak areas were derived and stored.

### Collection of the ecological factor data

Climate variables were obtained from the WorldClim database (https://www.worldclim.org), which contains data for 1971–2010 at a spatial resolution of 30 arc seconds. A total of 8 climate variables were extracted using ArcGIS 10.2 for each sample plot based on their geographic coordinates (latitude and longitude). These included annual average air temperature (°C), annual average highest temperature (°C), annual average lowest temperature (°C), annual average rainfall (mm), annual average relative humidity (%), average water vapor pressure, average air pressure and annual average sunshine hours. We also obtained soil samples using soil augers from three depths, namely, 0–10 cm, 10–20 cm, and 20–30 cm, with 3 replicates collected at each depth. These soil samples were analyzed for total N, available phosphorus and organic matter. The total nitrogen content was determined based on the semimicro Kjeldahl method using a nitrogen analyzer [29], available phosphorus (AP) was determined via the Mo-Sb colorimetric method [44], and organic matter (OM) was determined by the potassium dichromate volumetric method [45]. Other soil factor data were obtained from the World Soil Database (HWSD) and imported into ArcGIS software for extraction of sample data, including soil pH, organic carbon content, soil bulk density, clay content, sediment content, soil cation exchange capacity, and electrical conductivity. We also recorded geographical locations for each sampling point using a portable global positioning system (GPS) device (Beijing Huachen Beidou Information Technology Co., Ltd).

### Statistical analysis

All data were statistically analyzed using SPSS 26.0 software and are presented as the means ± SEM. Differences among groups were determined using one-way analysis of variance (ANOVA). RDA was performed using Canoco5 software, and the results were graphed using Origin Pro 2021 software.

### Electronic supplementary material

Below is the link to the electronic supplementary material.

Supplementary Material 1

## Data Availability

All data generated or analysed during this study are included in this published article. The datasets used and/or analysed during the current study are available from the corresponding author on reasonable request.
