# Supplementary material for: Metabolomics analysis reveals the metabolite profiles of Rheum tanguticum grown under different altitudinal gradients
Source: BMC Plant Biol. 2024 Mar 28;24:226. doi: 10.1186/s12870-024-04933-9 (PMC10976683; doi:10.1186/s12870-024-04933-9)
Supplement: Supplementary file 1 — Supplementary Material 1 [file 12870_2024_4933_MOESM1_ESM.doc]

**Supporting Information**

**Metabolomics analysis reveals metabolite profiles of *Rheum tanguticum* grown under different altitudinal gradients**

Lingling Wang ^1, 2, 3^, Shuo Zhao^1, 3^, Jianan Li^1, 3^, and Guoying Zhou ^1*^

1 Key Laboratory of Tibetan Medicine Research, Northwest Institute of Plateau Biology, Chinese Academy of Sciences, Xining 810008, China

2 Resource Institute for Chinese and Ethnic Materia Medica, Guizhou University of Traditional Chinese Medicine, Guiyang 550025, China

3 University of Chinese Academy of Sciences, Beijing 100049, China

* Correspondence: [zhougy@nwipb.cas](mailto:zhougy@nwipb.cas).cn


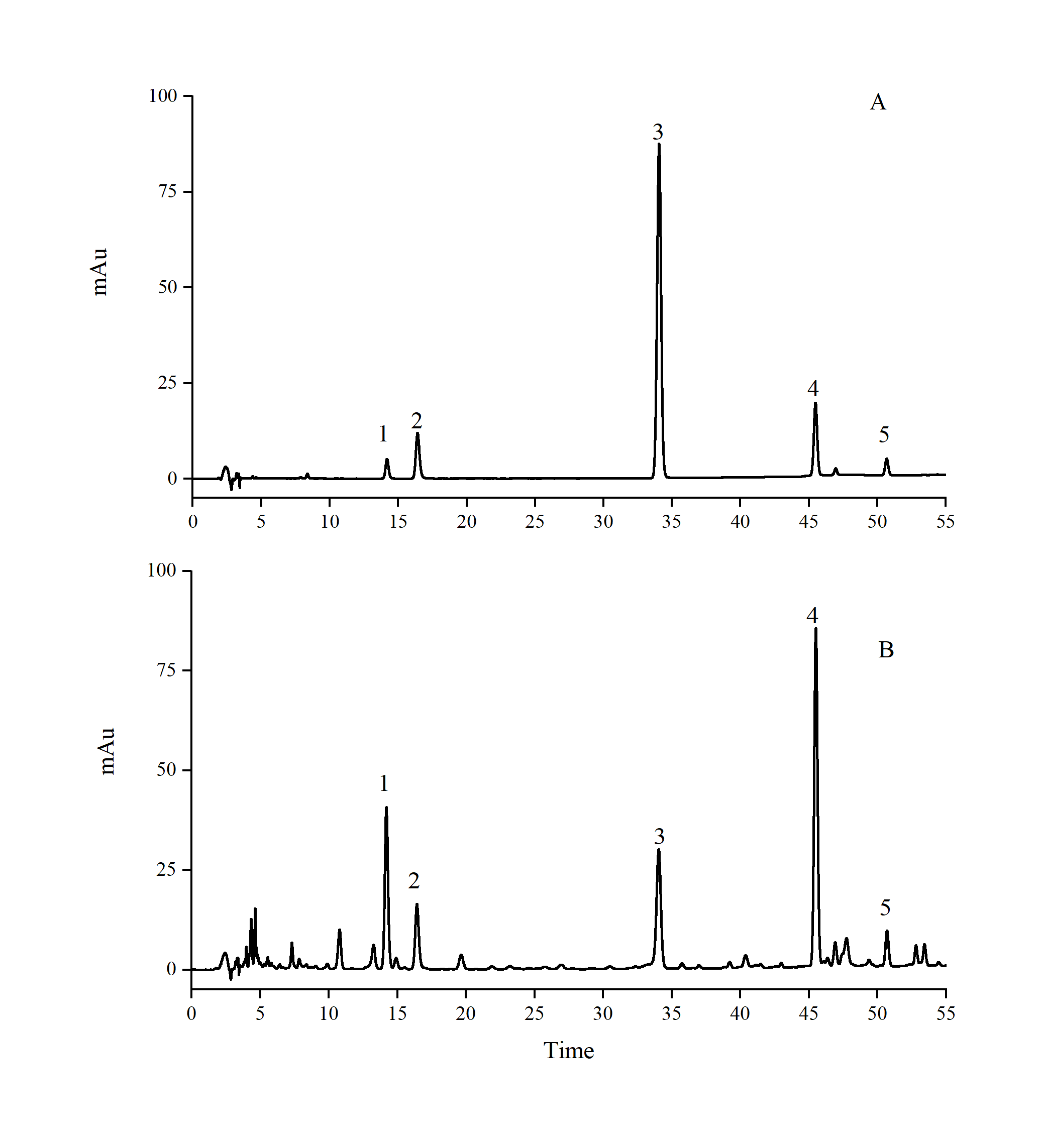


Fig. S1: Anthraquinone components chromatogram (A: standard, B: sample; 1: Aloe emodin, 2: rhein, 3: emodin, 4: chrysophanol, 5: emodin methyl ether).


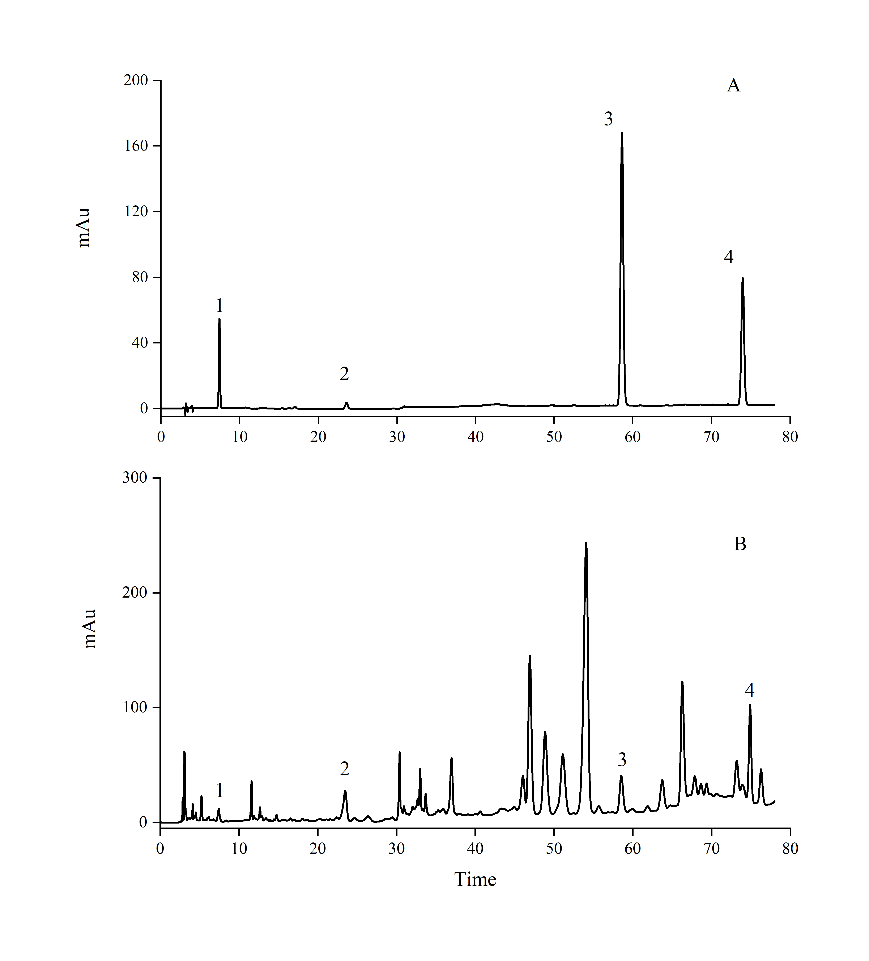


Fig. S2: Tannin and sennoside components chromatogram (A: standard, B: sample; 1: Gallic acid, 2: Catechin, 3: Sennoside B, 4: Sennoside A).
